# Spectrum of neurological disorders in neurology outpatients clinics in urban and rural Sindh, Pakistan: a cross sectional study

**DOI:** 10.1186/s12883-019-1424-1

**Published:** 2019-08-13

**Authors:** Safia Awan, Alam Ibrahim Siddiqi, Ahmed Asif, Naveeduddin Ahmed, Hazim Brohi, Sajad Jalbani, Mohammad Wasay

**Affiliations:** 1 Department of Medicine and Neurology, Aga Khan University, Karachi, Pakistan; 2 Department of Neurology, Shaheed Mohtarma Benazir Bhutto Medical University, Larkana, Pakistan; 3 Department of Neurology, Liaquat National Hospital, Karachi, Pakistan

**Keywords:** Neurological disorders, Stroke, Urban and rural population

## Abstract

**Background:**

Neurological disorders are the most common cause of morbidity and mortality in developing countries. Available evidence on urban–rural differences on neurological diseases is scare in such countries. Our study objective was to determine the prevalence of neurological diseases in urban and rural tertiary care hospitals of Sindh, Pakistan.

**Methods:**

This was a cross sectional study conducted in selected urban and rural region of tertiary care hospitals of Sindh, Pakistan. The outpatients medical records of adults (18 years and above) was obtained from January 1st, 2014 to December 31st, 2014.

**Results:**

A total of 10,786 outpatients visit were recorded in this period. Mean age of the participants was 40.6 ± 15 years; majority was females 6104 (56.6%). About three-fourth of the patients were from rural hospital 7828 (72.6%). Common neurological diseases were headache disorders 3613 (33.4%), nerve and root lesion 2928 (27.1%), vascular diseases 1440 (13.3%), epilepsies 566 (5.2%), muscle disorders 424 (3.9%), psychiatric disorders 340 (3.1%) and CNS infection 303 (2.8%). Comparison between the urban and rural samples showed that ischaemic stroke (72.7% vs. 82%) and psychiatric disorders (2.1% vs. 3.5%) were more prevalent in rural area as compared to urban setting.

**Conclusion:**

Stroke, headache and nerve and root lesion are major causes of neurological disorders in urban and rural settings of Sindh, Pakistan. The policy and planning must be focus on primary care, preventive measures and the promotion of health.

**Electronic supplementary material:**

The online version of this article (10.1186/s12883-019-1424-1) contains supplementary material, which is available to authorized users.

## Background

The demographic and epidemiological profile has changed the morbidity and mortality patterns worldwide. Neurological disorders are the fifth leading cause of death globally and are responsible for 5.53% of total global deaths [1]. Tension-type headache (1505·9 million cases), migraine (958·8 million), medication overuse headache (58·5 million), and Alzheimer’s disease including other dementias (46·0 million) were the most prevalent neurological disorders. Stroke is the leading cause of deaths accounting for 67.3% among all neurological disorders followed by Alzheimer’s disease and other dementias [2]. Death among young adults due to stroke increased significantly in developing countries and the number of disability-adjusted life years (DALYs) was seven times higher as compared to developed countries [3, 4]. The World Health Organization estimates that 80% of all strokes will occur in developing countries [5]. Dementia due to Alzheimer’s disease (AD) was more common 60% than vascular dementia 30% worldwide [6]. Epilepsy affects approximately 70 million people worldwide and 90% of the people suffering from epilepsy are in developing countries [7].

Stroke is a serious health concern in Pakistan with the annual incidence of 250/100,000 population [8–10]. The incidence of stroke was 21.8% (with 66.4% females) according to a study conducted in an urban slum of Karachi, Pakistan [11]. Epilepsy attacked 2.4 million people every year and it crippled 5 million for the rest of their life [12, 13]. The prevalence of depression and anxiety in Pakistan was 34% and it is more common among females. Depression is more prevalent in the rural areas (66% in women and 25% in men) than in urban areas (25% in women and 10% in men), [14–16]. Very limited data indicates that the prevalence of Alzheimer’s disease (AD) more frequent in elderly population. [17].

Information regarding the urban or rural profiles of neurological disorders obtained from hospitals or community from these areas is important [18]. It reflects the variation between rural and urban areas of the countries; it would also be the bases for policy changes and help to formulate large scale population based intervention studies. Study from India reported that the prevalence rate in urban and rural populations was 2190 and 4070/1,00,000 respectively [19]. A study conducted in tribal region of Uganda reported that the point prevalence of neurological diseases was 3.3% [20].

Epidemiological data regarding the neurological conditions is limited in Pakistan. Community based studies to determine the urban–rural differences of neurological diseases are very difficult to perform due to lack of resources, and because the number of neurologist is limited.

Previous work in Pakistan is based on specific diseases conditions and most were conducted in urban centers. The need would be to show the spectrum of neurological diseases and compare how their profile differs in urban and rural patients presenting to outpatient settings.

Therefore, our study objective was to determine the prevalence of neurological diseases in urban and rural tertiary care hospitals of Sindh, Pakistan. This dataset of different neurological diagnoses in urban and rural tertiary care hospitals serves as a starting point to inform policy and open up venue for further research. Sind is second largest province of Pakistan with a population of 47.89 Million.

## Methods

### Study design

This is a cross-sectional study conducted using outpatient medical records. Data on all adult (18 years and above) patients presenting to the selected outpatient clinic was obtained from January 2014 to December 2014.

### Study setting

The study was conducted in two tertiary care hospitals of the Sindh province of Pakistan representing urban and rural populations. Liaquat National Hospital & Medical College is a major semi-private hospital located in Karachi city that has a population of 14.9 million as recorded in the 2017 Census of Pakistan. Shaheed Mohtarma Benazir Bhutto Medical University (SMBBMU) is a public university of medicine, Nursing Health and medical sciences located in rural district of Larkana (population 1.5 Million).

### Data collection

The tool of the study has been reported previously and here we provide a brief summary [13]. A structured questionnaire was used to collect data on patient’s age, gender, place and type of residence (rural/urban), referral and patient’s evaluation status and diagnosis. The diagnosis was made by neurologist of the outpatient facility of selected hospitals. The neurological conditions were grouped according to International Classification of Disease [21] as follows: 1) Vascular diseases which include all type of stroke, moya moya disease, unruptured saccular aneurysm and arteriovenous malformation. 2) Cerebellar ataxia and hereditary spastic paraplegias include motor neuron diseases and sleep disorders. 3) Headache disorders 4) Acquired metabolic and toxic disorders which include all types of encephalopathy, psychosis, ataxia and delirium tremens. 5) CNS infections which include all type of meningitis, mucormycosis and herpes zoster. 6) CNS neoplasms, 7) Nerve and root lesion which includes neuritis, radiculitis, neuropathy, Guillain Barre Syndrome (GBS) and cranial nerve disorders. 8) Psychiatric disorders which include depression, conversion and bipolar disorder, anxiety neurosis, obsessive compulsive disorders, psychosis, and drug abuse. 9) Demyelinating disease 10) dementias 11) myopathies/muscle disorders, 12) developmental disorders 13) movement disorders, 14) epilepsies, 15) spinal disorders, and 16) neurometabolic/nutritional disorders of the nervous system.

### Statistical analysis

Descriptive analysis for demographic variables was performed; results are reported as numbers with percentages for quantitative variables. Continuous variables with normal and non-normal distributions are reported as mean (SD) and median [inter-quartile range (IQR)], respectively. Comparison between urban and rural population with neurological diseases were performed using Pearson Chi-square test and Continuous variables were compared using Student t test. All *p*-values were two sided and < 0.05 was considered as statistically significant. Data entry and data analysis was done by using SPSS 19.0 (SPSS Inc., Chicago: IL, USA).

## Results

A total of 10,786 outpatient visits were recorded across both sites during the study period. Mean age of the participants was 40.6 ± 15 years with a median age [IQR] of 40[29–50] years; 4682 (43.4%) males, 6104 (56.6%) females. Majority of the participants were from rural hospital 7828 (72.6%). Common neurological diseases were headache disorders 3613 (33.4%), nerve and root lesion 2928 (27.1%), vascular diseases 1440 (13.3%), epilepsies 566 (5.2%), muscle disorders 424 (3.9%), psychiatric disorders 340 (3.1%) and CNS infection 303 (2.8%); Table 1.

**Table 1:** Descriptive characteristics of study population (*n* = 10,786)

	n (%)
Age, in years	40.6 ± 15.0
Gender	
Male	4682 (43.4)
Female	6104 (56.6)
Residence	
Rural	7828 (72.6)
Urban	2958 (27.4)
Status (*n* = 10,776)	
New	6164 (57.2)
Follow-up	4612 (42.8)
Referral (*n* = 10,783)	
Self	2340 (21.7)
Physician	6166 (57.2)
Family/friends	2277 (21.1)
Vascular diseases (*n* = 1440)	
Stroke	1431/1440 (99.3%)
Ischaemic Stroke Acute/old	1141 (79.7)
Transient Ischaemic attack	37 (2.5)
Haemorrhagic stroke (parenchymal bleed)	253 (17.6)
Cerebellar Ataxias and Herediatory spactic paraplegias (*n* = 42)	
Motor Neuron diseases	28 (66.6)
Cerebellar ataxia	4 (9.5)
Cerebellar Degeneration	9 (21.4)
Headache Disorders (*n* = 3613)	
Migraine	957 (26.6)
Tension type headache	2631 (73)
Occipital Headache	14 (0.4)
Sleep disorders (*n* = 36)	
Narcolepsy	4 (11.1)
Obstructive sleep apnoea	5 (13.8)
Restless leg syndrome	27 (75.0)
Acquire Metabolic and Toxic Disorders (*n* = 8)	
CNS Infections (*n* = 303)	
Bactrial meningitis	156 (51.4)
Tuberculous meningitis / Tuberculoma	47 (15.5)
Brain abscess	11 (3.6)
Herpes zoster ophthalmicus/shingles	9 (2.9)
Tuberculoma	62 (20.4)
CNS Neoplasms (*n* = 35)	
Meningioma	10 (28.5)
Glioma	7 (0.2)
Astocytoma grade I-IV (GBM)	4 (11.4)
Pituitary adenoma/ Craniopharyngioma	5 (14.2)
Metastatic brain tumor	8 (22.8)
Nerve and root lesions (*n* = 2928)	
Guillain Barre Syndrome (GBS)	35 (1.1)
CIDP	57 (1.9)
Diabetic polyneuropathy	263 (8.9)
Cervical radiculopathy	761 (25.9)
Lumber radiculopathy	1589 (54.2)
Myopathies/Muscle Disorders (*n* = 424)	
Polymyositis	30 (7.0)
Myasthenia gravis	15 (3.5)
Fibromyalgia	12 (2.8)
Musculoskeltal pain	315 (74.2)
Hypokalemic periodic paralysis	12 (2.8)
Dementias (*n* = 194)	
Alzheimer’s diseases	116 (59.7)
Vascular dementia	60 (30.9)
Psychiatric Disorders (*n* = 340)	
Depression	171 (50.3)
Bipolar disorder	94 (27.6)
Anxiety neurosis	10 (2.9)
Obsessive compulsive disorders	15 (4.4)
Conversion disorders	32 (9.4)
Demyellinating Diseases (*n* = 59)	
Multiple sclerosis	29 (49.1)
Acute disseminated encephalomyelitis (ADEM)	8 (13.5)
Cerebellar Ataxias & Hereditary spastic paraplegias	6 (10.1)
Motor neurone diseases	9 (15.2)
Movement Disorders (*n* = 231)	
Parkinson’s diseases	154 (66.6)
Chorea	16 (6.9)
Tics	10 (4.3)
Dystonias	14 (6.0)
Cerebral palsy (CP)	13 (5.6)
Hemifacial spasm (HFS)	19 (8.2)
Epilepsies (*n* = 566)	
Focal Epilepsy	29 (5.1)
Generalized Epilepsy	529 (93.4)
Spinal Disorders (*n* = 156)	
Myelopathy (Spinal cord diseases)	124 (79.4)
Syringomyelia	14 (8.9)

Comparison between the rural and urban samples showed that Ischaemic stroke (82% vs. 72.7%) and psychiatric disorders (3.5% vs. 2.1%) were more prevalent in rural area as compare to urban population. However, headache disorders (32.1% vs. 35.3%), nerve and root lesions (28.5% vs. 26.6%) diabetes polyneuropathy (12.1% vs. 7.7%) and depression (73% vs. 45.1%) were comparatively higher in urban area as compare to rural area and this was the significant difference between the two populations Table 2.

**Table 2:** Comparison of neurological disorders in urban and rural areas (*n* = 10,786)

	Rural; *n* = 7828 n (%)	Urban; *n* = 2958 n (%)	*p* value
Age in years; mean ± SD	40.8 ± 15.2	40.2 ± 14.6	0.09
Gender			
Male	3635 (46.4)	1047 (35.4)	< 0.001
Female	4193 (53.6)	1911 (64.6)	
Stroke (*n* = 1431)			
Ischaemic stroke acute/old	891 (82)	250 (72.7)	< 0.001
Transient Ischaemic attack	19 (1.7)	18 (5.2)	
Haemorrhagic stroke	177 (16.3)	76 (22.1)	
Headache Disorders (*n* = 3613)			
Migraine	623 (24.6)	334 (31.2)	< 0.001
Tension type Headache	1900 (75)	731 (68.4)	
Occipital headache	10 (0.4)	4 (0.4)	
CNS infection (*n* = 303)	210 (2.7)	93 (3.1)	0.19
Meningitis; *n* = 210	144 (68.6)	66 (71)	0.67
Meningitis			
Bacterial Meningitis	107 (74.3)	49 (74.2)	0.47
Tuberculous/Tuberculoma	34 (23.6)	13 (19.7)	
Fungal meningitis	1 (0.7)	1 (1.5)	
Viral meningitis	2 (1.4)	3 (4.5)	
Nerve and root lesions; *n* = 2928	2084 (26.6)	844 (28.5)	0.04
Guillain Barre syndrome (AIDP)	26 (1.2)	9 (1.1)	0.68
CIDP	45 (2.2)	12 (1.4)	0.19
Diabetic polyneuropathy	161 (7.7)	102 (12.1)	< 0.001
Cervical radiculopathy	546 (26.2)	215 (25.5)	0.68
Lumber radiculopathy	1146 (55)	443 (52.5)	0.21
Psychiatric disorders; *n* = 340	277 (3.5)	63 (2.1)	< 0.001
Depression; *n* = 171	125 (45.1)	46 (73)	< 0.001
Alzheimer’s diseases; *n* = 116	86 (57.7)	30 (66.7)	0.28
Musculoskeletal pain; *n* = 315	225 (73.3)	90 (76.3)	0.52
Movement disorders; *n* = 221	167 (2.1)	54 (1.8)	0.30
Parkinsons; *n* = 150	117 (70.1)	33 (61.1)	0.22
Epilepsy; *n* = 566	408 (5.2)	158 (5.3)	0.78
Epilepsy type			
Focal Epilepsy	21 (5.1)	8 (5.1)	0.06
Generalized Epilepsy	361 (88.5)	148 (93.7)	
Myelopathy; *n* = 124	96 (80)	28 (77.8)	0.77
Spinal disorders; *n* = 156	120 (1.5)	36 (1.2)	0.24

Among the 7828 rural participants, the most prevalent neurological diagnoses were headache 1910 (24.4%), lumber radiculopathy 1146(14.6%), stroke 1087(13.9%), migraine 623(8.0%), cervical radiculopathy 546(7.0%) and epilepsy 408 (5.2%).

Among the 2958 urban participants, the most prevalent diagnoses were headache 735 (24.8%), lumber radiculopathy 443(15%), stroke 344(11.6%) and epilepsy 158(5.3%).

A stratification of participants by gender is represented in Table 3. Headache disorders were the most common type prevalent among females.

**Table 3:** neurological disorders and sex distribution among rural and urban population

	Rural; *n* = 7828		%	Urban; *n* = 2958		%
	Male; *n* = 3635	Female; *n* = 4193		Male; *n* = 1047	Female; 1911	
Headache disorders	752	1790	32.4	214	857	36.2
Nerve and root lesion	985	1099	26.6	319	525	28.5
Tension type headache	544	1356	24.2	146	585	24.7
Stroke	700	387	13.8	164	180	11.6

## Discussion

This is the first study to compare the neurological disorders among urban and rural population. The demographic characteristics of the study indicate that neurological disorders were more prevalent among women (56.6%) and in 30 to 50 years of age group. Previous study from Karachi reported the age group between 46 and 65 years which represented 34% of all out-patient neurological visits [13]. WHO, estimates show that 20% of adults aged 60 and over suffer from a mental or neurological disorder [22, 23]. A community based study from India reported the higher prevalence of neurological disorder among women as compared to men [19].

The first three prevalent disorders for both urban and rural areas are stroke, headache disorders and nerve and root lesions disorders. Almost similar trend was reported in previous studies [20, 24, 25]. A study from Karachi shows that the leading diagnoses were stroke 98.2%, Parkinson’s 67.7% and depression 77.5% [13].

A 72.5% (7828/10786) of participants was presented in the rural hospital as compared to 27.5% from urban center in our study. Only one government run tertiary care hospital is available to cater this population. The Basic Health Units and Rural dispensaries set up by the provincial and district governments in some villages, mostly have no doctors. Additionally, attracting and retaining well-qualified medical personnel is more challenging in rural environments.

Stroke is more prevalent in urban area but ischaemic stroke is more prevalent in rural area as compared to urban area. Most regions will see an increasing trend of stroke with the most notable rises in South Asia [26]. Similarly, stroke prevalence was higher in rural areas of India than in urban areas [27]. On the contrary, the incidence is higher in urban areas than in rural areas of Indonesia [28] and Thailand [29].

The overall prevalence of headache disorders was 33% and this is more prevalent in urban areas as compared to rural areas. This is slightly higher as compared to earlier study from India [30] but prevalence is higher among those from rural areas than urban [30]. Higher proportion of females is an almost consistent finding in many other studies [31].

The rate of neurologists in high income countries is 402 neurologists per 100,000 inhabitants while in low income countries it is only 4.5 per 100,000 inhabitants [32]. The number of beds for neurology patients is significantly lowered in low-income versus high-income regions in public hospitals (0.19/100,000 versus 11.73/100,000). In Pakistan, the rate is 1 neurologist per 1.5 million inhabitants [13, 33].

In Pakistan, most of the population doesn’t have access to neurology specialist in secondary and district level hospitals. The huge burden of patients and limited number of resources available in government hospitals made the situation worse for physicians. There is need to restructure the neurology training program. There is urgent need to develop a share model of teaching neurology program for general physicians and undergraduate level. Moreover, focus must be given to develop proper infrastructure and diagnostic facilities in the hospitals.

To our knowledge, this is the first study comparing rural and urban settings from Pakistan, Almost, 60.3% population in Pakistan lived in rural areas. Secondly, our study patients were evaluated and examination by neurologist. This study is not without limitations; this study was a hospital based and the fact that prevalence or incidence is better calculated by community-based prospective studies.

## Conclusion

Our study showed significant differences on different type of neurological disorders between the urban and rural populations. A headache disorder was the most frequent neurological condition in the rural population while stroke was more prevalent in urban population.

## Additional file

Additional file 1: Description of data: demographic and clinical characteristics. (DOCX 96 kb)

## Data Availability

The datasets generated and/or analysed during the current study are available from the corresponding author on reasonable request (Additional file 1).

## Abbreviations

AD: Alzheimer’s disease
AIDP: Guillain Barre Syndrome
DALYs: Disability-adjusted life years
NCDs: Neurological disorders
