# Supplementary material for: Spectrum of neurological disorders in neurology outpatients clinics in urban and rural Sindh, Pakistan: a cross sectional study
Source: BMC Neurol. 2019 Aug 13;19:192. doi: 10.1186/s12883-019-1424-1 (PMC6691523; doi:10.1186/s12883-019-1424-1)
Supplement: Supplementary file 1 — Description of data: demographic and clinical characteristics. (DOCX 96 kb) [file 12883_2019_1424_MOESM1_ESM.docx]

| **Statistics** | | |
| --- | --- | --- |
| Age | | |
| N | Valid | 10786 |
|  | Missing | 0 |
| Mean | | 40.66 |
| Std. Deviation | | 15.079 |

**Frequency Table**

| **age_cat** | | | | | |
| --- | --- | --- | --- | --- | --- |
|  | | Frequency | Percent | Valid Percent | Cumulative Percent |
| Valid | <30 | 3566 | 33.1 | 33.1 | 33.1 |
|  | 30-40 | 2617 | 24.3 | 24.3 | 57.3 |
|  | 41-50 | 2147 | 19.9 | 19.9 | 77.2 |
|  | 51-60 | 1486 | 13.8 | 13.8 | 91.0 |
|  | >60 | 970 | 9.0 | 9.0 | 100.0 |
|  | Total | 10786 | 100.0 | 100.0 |  |

| **City** | | | | | |
| --- | --- | --- | --- | --- | --- |
|  | | Frequency | Percent | Valid Percent | Cumulative Percent |
| Valid | Rural | 7828 | 72.6 | 72.6 | 72.6 |
|  | Urban | 2958 | 27.4 | 27.4 | 100.0 |
|  | Total | 10786 | 100.0 | 100.0 |  |

| **Gender** | | | | | |
| --- | --- | --- | --- | --- | --- |
|  | | Frequency | Percent | Valid Percent | Cumulative Percent |
| Valid | Male | 4682 | 43.4 | 43.4 | 43.4 |
|  | Female | 6104 | 56.6 | 56.6 | 100.0 |
|  | Total | 10786 | 100.0 | 100.0 |  |

| **stroke** | | | | | |
| --- | --- | --- | --- | --- | --- |
|  | | Frequency | Percent | Valid Percent | Cumulative Percent |
| Valid | No | 9 | .1 | .6 | .6 |
|  | Yes | 1431 | 13.3 | 99.4 | 100.0 |
|  | Total | 1440 | 13.4 | 100.0 |  |
| Missing | System | 9346 | 86.6 |  |  |
| Total | | 10786 | 100.0 |  |  |

| **Headache_dis** | | | | | |
| --- | --- | --- | --- | --- | --- |
|  | | Frequency | Percent | Valid Percent | Cumulative Percent |
| Valid | No | 7173 | 66.5 | 66.5 | 66.5 |
|  | Yes | 3613 | 33.5 | 33.5 | 100.0 |
|  | Total | 10786 | 100.0 | 100.0 |  |

| **type_head** | | | |
| --- | --- | --- | --- |
|  | | Frequency | Percent |
| Valid | Migraine | 957 | 8.9 |
|  | Tension type headache | 2631 | 24.4 |
|  | occipital headache | 14 | .1 |
|  | Total | 3602 | 33.4 |
| Missing | System | 7184 | 66.6 |
| Total | | 10786 | 100.0 |

| **type_head** | | | |
| --- | --- | --- | --- |
|  | | Valid Percent | Cumulative Percent |
| Valid | Migraine | 26.6 | 26.6 |
|  | Tension type headache | 73.0 | 99.6 |
|  | occipital headache | .4 | 100.0 |
|  | Total | 100.0 |  |
| Missing | System |  |  |
| Total | |  |  |

**Frequency Table**

| **nerve_root** | | | | | |
| --- | --- | --- | --- | --- | --- |
|  | | Frequency | Percent | Valid Percent | Cumulative Percent |
| Valid | No | 7858 | 72.9 | 72.9 | 72.9 |
|  | Yes | 2928 | 27.1 | 27.1 | 100.0 |
|  | Total | 10786 | 100.0 | 100.0 |  |

| **AIDP** | | | | | |
| --- | --- | --- | --- | --- | --- |
|  | | Frequency | Percent | Valid Percent | Cumulative Percent |
| Valid | No | 2893 | 26.8 | 98.8 | 98.8 |
|  | Yes | 35 | .3 | 1.2 | 100.0 |
|  | Total | 2928 | 27.1 | 100.0 |  |
| Missing | System | 7858 | 72.9 |  |  |
| Total | | 10786 | 100.0 |  |  |

| **CIDP** | | | | | |
| --- | --- | --- | --- | --- | --- |
|  | | Frequency | Percent | Valid Percent | Cumulative Percent |
| Valid | No | 2871 | 26.6 | 98.1 | 98.1 |
|  | Yes | 57 | .5 | 1.9 | 100.0 |
|  | Total | 2928 | 27.1 | 100.0 |  |
| Missing | System | 7858 | 72.9 |  |  |
| Total | | 10786 | 100.0 |  |  |

| **Diabetic polyneuropathy** | | | | | |
| --- | --- | --- | --- | --- | --- |
|  | | Frequency | Percent | Valid Percent | Cumulative Percent |
| Valid | No | 2665 | 24.7 | 91.0 | 91.0 |
|  | Yes | 263 | 2.4 | 9.0 | 100.0 |
|  | Total | 2928 | 27.1 | 100.0 |  |
| Missing | System | 7858 | 72.9 |  |  |
| Total | | 10786 | 100.0 |  |  |

| **Cervical radiculopathy** | | | | | |
| --- | --- | --- | --- | --- | --- |
|  | | Frequency | Percent | Valid Percent | Cumulative Percent |
| Valid | No | 2167 | 20.1 | 74.0 | 74.0 |
|  | Yes | 761 | 7.1 | 26.0 | 100.0 |
|  | Total | 2928 | 27.1 | 100.0 |  |
| Missing | System | 7858 | 72.9 |  |  |
| Total | | 10786 | 100.0 |  |  |

| **Lumber radiculopathy** | | | | | |
| --- | --- | --- | --- | --- | --- |
|  | | Frequency | Percent | Valid Percent | Cumulative Percent |
| Valid | 0 | 1339 | 12.4 | 45.7 | 45.7 |
|  | Yes | 1589 | 14.7 | 54.3 | 100.0 |
|  | Total | 2928 | 27.1 | 100.0 |  |
| Missing | System | 7858 | 72.9 |  |  |
| Total | | 10786 | 100.0 |  |  |

| **psychatric** | | | | | |
| --- | --- | --- | --- | --- | --- |
|  | | Frequency | Percent | Valid Percent | Cumulative Percent |
| Valid | 0 | 10446 | 96.8 | 96.8 | 96.8 |
|  | 1 | 340 | 3.2 | 3.2 | 100.0 |
|  | Total | 10786 | 100.0 | 100.0 |  |

| **depression** | | | | | |
| --- | --- | --- | --- | --- | --- |
|  | | Frequency | Percent | Valid Percent | Cumulative Percent |
| Valid | No | 169 | 1.6 | 49.7 | 49.7 |
|  | Yes | 171 | 1.6 | 50.3 | 100.0 |
|  | Total | 340 | 3.2 | 100.0 |  |
| Missing | System | 10446 | 96.8 |  |  |
| Total | | 10786 | 100.0 |  |  |

| **alzheimer** | | | | | |
| --- | --- | --- | --- | --- | --- |
|  | | Frequency | Percent | Valid Percent | Cumulative Percent |
| Valid | No | 78 | .7 | 40.2 | 40.2 |
|  | Yes | 116 | 1.1 | 59.8 | 100.0 |
|  | Total | 194 | 1.8 | 100.0 |  |
| Missing | System | 10592 | 98.2 |  |  |
| Total | | 10786 | 100.0 |  |  |

SORT CASES BY City.

| **stroke * Gender Crosstabulation** | | | | | | |
| --- | --- | --- | --- | --- | --- | --- |
| City | | | | Gender | | Total |
|  |  |  |  | Male | Female |  |
| Rural | stroke | No | Count | 4 | 5 | 9 |
|  |  |  | % within Gender | .6% | 1.3% | .8% |
|  |  | Yes | Count | 700 | 387 | 1087 |
|  |  |  | % within Gender | 99.4% | 98.7% | 99.2% |
|  | Total | | Count | 704 | 392 | 1096 |
|  |  |  | % within Gender | 100.0% | 100.0% | 100.0% |
| Urban | stroke | Yes | Count | 164 | 180 | 344 |
|  |  |  | % within Gender | 100.0% | 100.0% | 100.0% |
|  | Total | | Count | 164 | 180 | 344 |
|  |  |  | % within Gender | 100.0% | 100.0% | 100.0% |

| **Headache_dis * Gender Crosstabulation** | | | | | | |
| --- | --- | --- | --- | --- | --- | --- |
| City | | | | Gender | | Total |
|  |  |  |  | Male | Female |  |
| Rural | Headache_dis | No | Count | 2883 | 2403 | 5286 |
|  |  |  | % within Gender | 79.3% | 57.3% | 67.5% |
|  |  | Yes | Count | 752 | 1790 | 2542 |
|  |  |  | % within Gender | 20.7% | 42.7% | 32.5% |
|  | Total | | Count | 3635 | 4193 | 7828 |
|  |  |  | % within Gender | 100.0% | 100.0% | 100.0% |
| Urban | Headache_dis | No | Count | 833 | 1054 | 1887 |
|  |  |  | % within Gender | 79.6% | 55.2% | 63.8% |
|  |  | Yes | Count | 214 | 857 | 1071 |
|  |  |  | % within Gender | 20.4% | 44.8% | 36.2% |
|  | Total | | Count | 1047 | 1911 | 2958 |
|  |  |  | % within Gender | 100.0% | 100.0% | 100.0% |

| **nerve_root * Gender Crosstabulation** | | | | | | |
| --- | --- | --- | --- | --- | --- | --- |
| City | | | | Gender | | Total |
|  |  |  |  | Male | Female |  |
| Rural | nerve_root | No | Count | 2650 | 3094 | 5744 |
|  |  |  | % within Gender | 72.9% | 73.8% | 73.4% |
|  |  | Yes | Count | 985 | 1099 | 2084 |
|  |  |  | % within Gender | 27.1% | 26.2% | 26.6% |
|  | Total | | Count | 3635 | 4193 | 7828 |
|  |  |  | % within Gender | 100.0% | 100.0% | 100.0% |
| Urban | nerve_root | No | Count | 728 | 1386 | 2114 |
|  |  |  | % within Gender | 69.5% | 72.5% | 71.5% |
|  |  | Yes | Count | 319 | 525 | 844 |
|  |  |  | % within Gender | 30.5% | 27.5% | 28.5% |
|  | Total | | Count | 1047 | 1911 | 2958 |
|  |  |  | % within Gender | 100.0% | 100.0% | 100.0% |

| **type_head * Gender Crosstabulation** | | | | | |
| --- | --- | --- | --- | --- | --- |
| City | | | | Gender | |
|  |  |  |  | Male | Female |
| Rural | type_head | Migraine | Count | 198 | 425 |
|  |  |  | % within Gender | 26.4% | 23.8% |
|  |  | Tension type headache | Count | 544 | 1356 |
|  |  |  | % within Gender | 72.6% | 76.0% |
|  |  | occipital headache | Count | 7 | 3 |
|  |  |  | % within Gender | .9% | .2% |
|  | Total | | Count | 749 | 1784 |
|  |  |  | % within Gender | 100.0% | 100.0% |
| Urban | type_head | Migraine | Count | 66 | 268 |
|  |  |  | % within Gender | 30.8% | 31.3% |
|  |  | Tension type headache | Count | 146 | 585 |
|  |  |  | % within Gender | 68.2% | 68.4% |
|  |  | occipital headache | Count | 2 | 2 |
|  |  |  | % within Gender | .9% | .2% |
|  | Total | | Count | 214 | 855 |
|  |  |  | % within Gender | 100.0% | 100.0% |

| **type_head * Gender Crosstabulation** | | | | |
| --- | --- | --- | --- | --- |
| City | | | | Total |
| Rural | type_head | Migraine | Count | 623 |
|  |  |  | % within Gender | 24.6% |
|  |  | Tension type headache | Count | 1900 |
|  |  |  | % within Gender | 75.0% |
|  |  | occipital headache | Count | 10 |
|  |  |  | % within Gender | .4% |
|  | Total | | Count | 2533 |
|  |  |  | % within Gender | 100.0% |
| Urban | type_head | Migraine | Count | 334 |
|  |  |  | % within Gender | 31.2% |
|  |  | Tension type headache | Count | 731 |
|  |  |  | % within Gender | 68.4% |
|  |  | occipital headache | Count | 4 |
|  |  |  | % within Gender | .4% |
|  | Total | | Count | 1069 |
|  |  |  | % within Gender | 100.0% |

**Gender * City**

| **Crosstab** | | | | | |
| --- | --- | --- | --- | --- | --- |
|  | | | City | | Total |
|  |  |  | Rural | Urban |  |
| Gender | Male | Count | 3635 | 1047 | 4682 |
|  |  | % within City | 46.4% | 35.4% | 43.4% |
|  | Female | Count | 4193 | 1911 | 6104 |
|  |  | % within City | 53.6% | 64.6% | 56.6% |
| Total | | Count | 7828 | 2958 | 10786 |
|  |  | % within City | 100.0% | 100.0% | 100.0% |

| **Chi-Square Tests** | | | |
| --- | --- | --- | --- |
|  | Value | df | Asymp. Sig. (2-sided) |
| Pearson Chi-Square | 106.519^a^ | 1 | .000 |
| Continuity Correction^b^ | 106.070 | 1 | .000 |
| Likelihood Ratio | 107.871 | 1 | .000 |
| Fisher's Exact Test |  |  |  |
| Linear-by-Linear Association | 106.509 | 1 | .000 |
| N of Valid Cases | 10786 |  |  |
| a. 0 cells (.0%) have expected count less than 5. The minimum expected count is 1284.01. | | | |
| b. Computed only for a 2x2 table | | | |

| **Chi-Square Tests** | | |
| --- | --- | --- |
|  | Exact Sig. (2-sided) | Exact Sig. (1-sided) |
| Pearson Chi-Square |  |  |
| Continuity Correction^b^ |  |  |
| Likelihood Ratio |  |  |
| Fisher's Exact Test | .000 | .000 |
| Linear-by-Linear Association |  |  |
| N of Valid Cases |  |  |
|  | | |
| b. Computed only for a 2x2 table | | |

**stroke * City**

| **Crosstab** | | | | | |
| --- | --- | --- | --- | --- | --- |
|  | | | City | | Total |
|  |  |  | Rural | Urban |  |
| stroke | No | Count | 9 | 0 | 9 |
|  |  | % within City | .8% | .0% | .6% |
|  | Yes | Count | 1087 | 344 | 1431 |
|  |  | % within City | 99.2% | 100.0% | 99.4% |
| Total | | Count | 1096 | 344 | 1440 |
|  |  | % within City | 100.0% | 100.0% | 100.0% |

| **Chi-Square Tests** | | | |
| --- | --- | --- | --- |
|  | Value | df | Asymp. Sig. (2-sided) |
| Pearson Chi-Square | 2.843^a^ | 1 | .092 |
| Continuity Correction^b^ | 1.674 | 1 | .196 |
| Likelihood Ratio | 4.931 | 1 | .026 |
| Fisher's Exact Test |  |  |  |
| Linear-by-Linear Association | 2.841 | 1 | .092 |
| N of Valid Cases | 1440 |  |  |
| a. 1 cells (25.0%) have expected count less than 5. The minimum expected count is 2.15. | | | |
| b. Computed only for a 2x2 table | | | |

| **Chi-Square Tests** | | |
| --- | --- | --- |
|  | Exact Sig. (2-sided) | Exact Sig. (1-sided) |
| Pearson Chi-Square |  |  |
| Continuity Correction^b^ |  |  |
| Likelihood Ratio |  |  |
| Fisher's Exact Test | .125 | .085 |
| Linear-by-Linear Association |  |  |
| N of Valid Cases |  |  |
|  | | |
| b. Computed only for a 2x2 table | | |

**Stroke_t * City**

| **Crosstab** | | | | | |
| --- | --- | --- | --- | --- | --- |
|  | | | City | | Total |
|  |  |  | Rural | Urban |  |
| Stroke_t | Ischaemic acte/old | Count | 891 | 250 | 1141 |
|  |  | % within City | 82.0% | 72.7% | 79.7% |
|  | Transient | Count | 19 | 18 | 37 |
|  |  | % within City | 1.7% | 5.2% | 2.6% |
|  | Hemorrhagic | Count | 177 | 76 | 253 |
|  |  | % within City | 16.3% | 22.1% | 17.7% |
| Total | | Count | 1087 | 344 | 1431 |
|  |  | % within City | 100.0% | 100.0% | 100.0% |

| **Chi-Square Tests** | | | |
| --- | --- | --- | --- |
|  | Value | df | Asymp. Sig. (2-sided) |
| Pearson Chi-Square | 20.091^a^ | 2 | .000 |
| Likelihood Ratio | 18.131 | 2 | .000 |
| Linear-by-Linear Association | 10.113 | 1 | .001 |
| N of Valid Cases | 1431 |  |  |
| a. 0 cells (.0%) have expected count less than 5. The minimum expected count is 8.89. | | | |

**Headache_dis * City**

| **Crosstab** | | | | | |
| --- | --- | --- | --- | --- | --- |
|  | | | City | | Total |
|  |  |  | Rural | Urban |  |
| Headache_dis | No | Count | 5286 | 1887 | 7173 |
|  |  | % within City | 67.5% | 63.8% | 66.5% |
|  | Yes | Count | 2542 | 1071 | 3613 |
|  |  | % within City | 32.5% | 36.2% | 33.5% |
| Total | | Count | 7828 | 2958 | 10786 |
|  |  | % within City | 100.0% | 100.0% | 100.0% |

| **Chi-Square Tests** | | | |
| --- | --- | --- | --- |
|  | Value | df | Asymp. Sig. (2-sided) |
| Pearson Chi-Square | 13.435^a^ | 1 | .000 |
| Continuity Correction^b^ | 13.268 | 1 | .000 |
| Likelihood Ratio | 13.331 | 1 | .000 |
| Fisher's Exact Test |  |  |  |
| Linear-by-Linear Association | 13.433 | 1 | .000 |
| N of Valid Cases | 10786 |  |  |
| a. 0 cells (.0%) have expected count less than 5. The minimum expected count is 990.84. | | | |
| b. Computed only for a 2x2 table | | | |

| **Chi-Square Tests** | | |
| --- | --- | --- |
|  | Exact Sig. (2-sided) | Exact Sig. (1-sided) |
| Pearson Chi-Square |  |  |
| Continuity Correction^b^ |  |  |
| Likelihood Ratio |  |  |
| Fisher's Exact Test | .000 | .000 |
| Linear-by-Linear Association |  |  |
| N of Valid Cases |  |  |
|  | | |
| b. Computed only for a 2x2 table | | |

**CNSinfec * City**

| **Crosstab** | | | | | |
| --- | --- | --- | --- | --- | --- |
|  | | | City | | Total |
|  |  |  | Rural | Urban |  |
| CNSinfec | No | Count | 7618 | 2865 | 10483 |
|  |  | % within City | 97.3% | 96.9% | 97.2% |
|  | Yes | Count | 210 | 93 | 303 |
|  |  | % within City | 2.7% | 3.1% | 2.8% |
| Total | | Count | 7828 | 2958 | 10786 |
|  |  | % within City | 100.0% | 100.0% | 100.0% |

| **Chi-Square Tests** | | | |
| --- | --- | --- | --- |
|  | Value | df | Asymp. Sig. (2-sided) |
| Pearson Chi-Square | 1.673^a^ | 1 | .196 |
| Continuity Correction^b^ | 1.509 | 1 | .219 |
| Likelihood Ratio | 1.636 | 1 | .201 |
| Fisher's Exact Test |  |  |  |
| Linear-by-Linear Association | 1.673 | 1 | .196 |
| N of Valid Cases | 10786 |  |  |
| a. 0 cells (.0%) have expected count less than 5. The minimum expected count is 83.10. | | | |
| b. Computed only for a 2x2 table | | | |

| **Chi-Square Tests** | | |
| --- | --- | --- |
|  | Exact Sig. (2-sided) | Exact Sig. (1-sided) |
| Pearson Chi-Square |  |  |
| Continuity Correction^b^ |  |  |
| Likelihood Ratio |  |  |
| Fisher's Exact Test | .214 | .110 |
| Linear-by-Linear Association |  |  |
| N of Valid Cases |  |  |
|  | | |
| b. Computed only for a 2x2 table | | |

**meningitis * City**

| **Crosstab** | | | | | |
| --- | --- | --- | --- | --- | --- |
|  | | | City | | Total |
|  |  |  | Rural | Urban |  |
| meningitis | No | Count | 66 | 27 | 93 |
|  |  | % within City | 31.4% | 29.0% | 30.7% |
|  | Yes | Count | 144 | 66 | 210 |
|  |  | % within City | 68.6% | 71.0% | 69.3% |
| Total | | Count | 210 | 93 | 303 |
|  |  | % within City | 100.0% | 100.0% | 100.0% |

| **Chi-Square Tests** | | | |
| --- | --- | --- | --- |
|  | Value | df | Asymp. Sig. (2-sided) |
| Pearson Chi-Square | .174^a^ | 1 | .677 |
| Continuity Correction^b^ | .080 | 1 | .778 |
| Likelihood Ratio | .175 | 1 | .676 |
| Fisher's Exact Test |  |  |  |
| Linear-by-Linear Association | .173 | 1 | .677 |
| N of Valid Cases | 303 |  |  |
| a. 0 cells (.0%) have expected count less than 5. The minimum expected count is 28.54. | | | |
| b. Computed only for a 2x2 table | | | |

| **Chi-Square Tests** | | |
| --- | --- | --- |
|  | Exact Sig. (2-sided) | Exact Sig. (1-sided) |
| Pearson Chi-Square |  |  |
| Continuity Correction^b^ |  |  |
| Likelihood Ratio |  |  |
| Fisher's Exact Test | .787 | .391 |
| Linear-by-Linear Association |  |  |
| N of Valid Cases |  |  |
|  | | |
| b. Computed only for a 2x2 table | | |

**nerve_root * City**

| **Crosstab** | | | | | |
| --- | --- | --- | --- | --- | --- |
|  | | | City | | Total |
|  |  |  | Rural | Urban |  |
| nerve_root | No | Count | 5744 | 2114 | 7858 |
|  |  | % within City | 73.4% | 71.5% | 72.9% |
|  | Yes | Count | 2084 | 844 | 2928 |
|  |  | % within City | 26.6% | 28.5% | 27.1% |
| Total | | Count | 7828 | 2958 | 10786 |
|  |  | % within City | 100.0% | 100.0% | 100.0% |

| **Chi-Square Tests** | | | |
| --- | --- | --- | --- |
|  | Value | df | Asymp. Sig. (2-sided) |
| Pearson Chi-Square | 3.962^a^ | 1 | .047 |
| Continuity Correction^b^ | 3.866 | 1 | .049 |
| Likelihood Ratio | 3.936 | 1 | .047 |
| Fisher's Exact Test |  |  |  |
| Linear-by-Linear Association | 3.961 | 1 | .047 |
| N of Valid Cases | 10786 |  |  |
| a. 0 cells (.0%) have expected count less than 5. The minimum expected count is 802.99. | | | |
| b. Computed only for a 2x2 table | | | |

| **Chi-Square Tests** | | |
| --- | --- | --- |
|  | Exact Sig. (2-sided) | Exact Sig. (1-sided) |
| Pearson Chi-Square |  |  |
| Continuity Correction^b^ |  |  |
| Likelihood Ratio |  |  |
| Fisher's Exact Test | .049 | .025 |
| Linear-by-Linear Association |  |  |
| N of Valid Cases |  |  |
|  | | |
| b. Computed only for a 2x2 table | | |

**AIDP * City**

| **Crosstab** | | | | | |
| --- | --- | --- | --- | --- | --- |
|  | | | City | | Total |
|  |  |  | Rural | Urban |  |
| AIDP | No | Count | 2058 | 835 | 2893 |
|  |  | % within City | 98.8% | 98.9% | 98.8% |
|  | Yes | Count | 26 | 9 | 35 |
|  |  | % within City | 1.2% | 1.1% | 1.2% |
| Total | | Count | 2084 | 844 | 2928 |
|  |  | % within City | 100.0% | 100.0% | 100.0% |

| **Chi-Square Tests** | | | |
| --- | --- | --- | --- |
|  | Value | df | Asymp. Sig. (2-sided) |
| Pearson Chi-Square | .167^a^ | 1 | .683 |
| Continuity Correction^b^ | .049 | 1 | .825 |
| Likelihood Ratio | .171 | 1 | .679 |
| Fisher's Exact Test |  |  |  |
| Linear-by-Linear Association | .167 | 1 | .683 |
| N of Valid Cases | 2928 |  |  |
| a. 0 cells (.0%) have expected count less than 5. The minimum expected count is 10.09. | | | |
| b. Computed only for a 2x2 table | | | |

| **Chi-Square Tests** | | |
| --- | --- | --- |
|  | Exact Sig. (2-sided) | Exact Sig. (1-sided) |
| Pearson Chi-Square |  |  |
| Continuity Correction^b^ |  |  |
| Likelihood Ratio |  |  |
| Fisher's Exact Test | .851 | .423 |
| Linear-by-Linear Association |  |  |
| N of Valid Cases |  |  |
|  | | |
| b. Computed only for a 2x2 table | | |

**CIDP * City**

| **Crosstab** | | | | | |
| --- | --- | --- | --- | --- | --- |
|  | | | City | | Total |
|  |  |  | Rural | Urban |  |
| CIDP | No | Count | 2039 | 832 | 2871 |
|  |  | % within City | 97.8% | 98.6% | 98.1% |
|  | Yes | Count | 45 | 12 | 57 |
|  |  | % within City | 2.2% | 1.4% | 1.9% |
| Total | | Count | 2084 | 844 | 2928 |
|  |  | % within City | 100.0% | 100.0% | 100.0% |

| **Chi-Square Tests** | | | |
| --- | --- | --- | --- |
|  | Value | df | Asymp. Sig. (2-sided) |
| Pearson Chi-Square | 1.712^a^ | 1 | .191 |
| Continuity Correction^b^ | 1.347 | 1 | .246 |
| Likelihood Ratio | 1.820 | 1 | .177 |
| Fisher's Exact Test |  |  |  |
| Linear-by-Linear Association | 1.711 | 1 | .191 |
| N of Valid Cases | 2928 |  |  |
| a. 0 cells (.0%) have expected count less than 5. The minimum expected count is 16.43. | | | |
| b. Computed only for a 2x2 table | | | |

| **Chi-Square Tests** | | |
| --- | --- | --- |
|  | Exact Sig. (2-sided) | Exact Sig. (1-sided) |
| Pearson Chi-Square |  |  |
| Continuity Correction^b^ |  |  |
| Likelihood Ratio |  |  |
| Fisher's Exact Test | .237 | .121 |
| Linear-by-Linear Association |  |  |
| N of Valid Cases |  |  |
|  | | |
| b. Computed only for a 2x2 table | | |

**Diabetic polyneuropathy * City**

| **Crosstab** | | | | | |
| --- | --- | --- | --- | --- | --- |
|  | | | City | | Total |
|  |  |  | Rural | Urban |  |
| Diabetic polyneuropathy | No | Count | 1923 | 742 | 2665 |
|  |  | % within City | 92.3% | 87.9% | 91.0% |
|  | Yes | Count | 161 | 102 | 263 |
|  |  | % within City | 7.7% | 12.1% | 9.0% |
| Total | | Count | 2084 | 844 | 2928 |
|  |  | % within City | 100.0% | 100.0% | 100.0% |

| **Chi-Square Tests** | | | |
| --- | --- | --- | --- |
|  | Value | df | Asymp. Sig. (2-sided) |
| Pearson Chi-Square | 13.966^a^ | 1 | .000 |
| Continuity Correction^b^ | 13.438 | 1 | .000 |
| Likelihood Ratio | 13.267 | 1 | .000 |
| Fisher's Exact Test |  |  |  |
| Linear-by-Linear Association | 13.962 | 1 | .000 |
| N of Valid Cases | 2928 |  |  |
| a. 0 cells (.0%) have expected count less than 5. The minimum expected count is 75.81. | | | |
| b. Computed only for a 2x2 table | | | |

| **Chi-Square Tests** | | |
| --- | --- | --- |
|  | Exact Sig. (2-sided) | Exact Sig. (1-sided) |
| Pearson Chi-Square |  |  |
| Continuity Correction^b^ |  |  |
| Likelihood Ratio |  |  |
| Fisher's Exact Test | .000 | .000 |
| Linear-by-Linear Association |  |  |
| N of Valid Cases |  |  |
|  | | |
| b. Computed only for a 2x2 table | | |

**Cervical radiculopathy * City**

| **Crosstab** | | | | | |
| --- | --- | --- | --- | --- | --- |
|  | | | City | | Total |
|  |  |  | Rural | Urban |  |
| Cervical radiculopathy | No | Count | 1538 | 629 | 2167 |
|  |  | % within City | 73.8% | 74.5% | 74.0% |
|  | Yes | Count | 546 | 215 | 761 |
|  |  | % within City | 26.2% | 25.5% | 26.0% |
| Total | | Count | 2084 | 844 | 2928 |
|  |  | % within City | 100.0% | 100.0% | 100.0% |

| **Chi-Square Tests** | | | |
| --- | --- | --- | --- |
|  | Value | df | Asymp. Sig. (2-sided) |
| Pearson Chi-Square | .164^a^ | 1 | .685 |
| Continuity Correction^b^ | .129 | 1 | .720 |
| Likelihood Ratio | .165 | 1 | .685 |
| Fisher's Exact Test |  |  |  |
| Linear-by-Linear Association | .164 | 1 | .685 |
| N of Valid Cases | 2928 |  |  |
| a. 0 cells (.0%) have expected count less than 5. The minimum expected count is 219.36. | | | |
| b. Computed only for a 2x2 table | | | |

| **Chi-Square Tests** | | |
| --- | --- | --- |
|  | Exact Sig. (2-sided) | Exact Sig. (1-sided) |
| Pearson Chi-Square |  |  |
| Continuity Correction^b^ |  |  |
| Likelihood Ratio |  |  |
| Fisher's Exact Test | .710 | .361 |
| Linear-by-Linear Association |  |  |
| N of Valid Cases |  |  |
|  | | |
| b. Computed only for a 2x2 table | | |

**Lumber radiculopathy * City**

| **Crosstab** | | | | | |
| --- | --- | --- | --- | --- | --- |
|  | | | City | | Total |
|  |  |  | Rural | Urban |  |
| Lumber radiculopathy | 0 | Count | 938 | 401 | 1339 |
|  |  | % within City | 45.0% | 47.5% | 45.7% |
|  | Yes | Count | 1146 | 443 | 1589 |
|  |  | % within City | 55.0% | 52.5% | 54.3% |
| Total | | Count | 2084 | 844 | 2928 |
|  |  | % within City | 100.0% | 100.0% | 100.0% |

| **Chi-Square Tests** | | | |
| --- | --- | --- | --- |
|  | Value | df | Asymp. Sig. (2-sided) |
| Pearson Chi-Square | 1.516^a^ | 1 | .218 |
| Continuity Correction^b^ | 1.416 | 1 | .234 |
| Likelihood Ratio | 1.514 | 1 | .219 |
| Fisher's Exact Test |  |  |  |
| Linear-by-Linear Association | 1.515 | 1 | .218 |
| N of Valid Cases | 2928 |  |  |
| a. 0 cells (.0%) have expected count less than 5. The minimum expected count is 385.97. | | | |
| b. Computed only for a 2x2 table | | | |

| **Chi-Square Tests** | | |
| --- | --- | --- |
|  | Exact Sig. (2-sided) | Exact Sig. (1-sided) |
| Pearson Chi-Square |  |  |
| Continuity Correction^b^ |  |  |
| Likelihood Ratio |  |  |
| Fisher's Exact Test | .220 | .117 |
| Linear-by-Linear Association |  |  |
| N of Valid Cases |  |  |
|  | | |
| b. Computed only for a 2x2 table | | |

**psychatric * City**

| **Crosstab** | | | | | |
| --- | --- | --- | --- | --- | --- |
|  | | | City | | Total |
|  |  |  | Rural | Urban |  |
| psychatric | 0 | Count | 7551 | 2895 | 10446 |
|  |  | % within City | 96.5% | 97.9% | 96.8% |
|  | 1 | Count | 277 | 63 | 340 |
|  |  | % within City | 3.5% | 2.1% | 3.2% |
| Total | | Count | 7828 | 2958 | 10786 |
|  |  | % within City | 100.0% | 100.0% | 100.0% |

| **Chi-Square Tests** | | | |
| --- | --- | --- | --- |
|  | Value | df | Asymp. Sig. (2-sided) |
| Pearson Chi-Square | 13.956^a^ | 1 | .000 |
| Continuity Correction^b^ | 13.498 | 1 | .000 |
| Likelihood Ratio | 15.088 | 1 | .000 |
| Fisher's Exact Test |  |  |  |
| Linear-by-Linear Association | 13.955 | 1 | .000 |
| N of Valid Cases | 10786 |  |  |
| a. 0 cells (.0%) have expected count less than 5. The minimum expected count is 93.24. | | | |
| b. Computed only for a 2x2 table | | | |

| **Chi-Square Tests** | | |
| --- | --- | --- |
|  | Exact Sig. (2-sided) | Exact Sig. (1-sided) |
| Pearson Chi-Square |  |  |
| Continuity Correction^b^ |  |  |
| Likelihood Ratio |  |  |
| Fisher's Exact Test | .000 | .000 |
| Linear-by-Linear Association |  |  |
| N of Valid Cases |  |  |
|  | | |
| b. Computed only for a 2x2 table | | |

**depression * City**

| **Crosstab** | | | | | |
| --- | --- | --- | --- | --- | --- |
|  | | | City | | Total |
|  |  |  | Rural | Urban |  |
| depression | No | Count | 152 | 17 | 169 |
|  |  | % within City | 54.9% | 27.0% | 49.7% |
|  | Yes | Count | 125 | 46 | 171 |
|  |  | % within City | 45.1% | 73.0% | 50.3% |
| Total | | Count | 277 | 63 | 340 |
|  |  | % within City | 100.0% | 100.0% | 100.0% |

| **Chi-Square Tests** | | | |
| --- | --- | --- | --- |
|  | Value | df | Asymp. Sig. (2-sided) |
| Pearson Chi-Square | 15.970^a^ | 1 | .000 |
| Continuity Correction^b^ | 14.874 | 1 | .000 |
| Likelihood Ratio | 16.490 | 1 | .000 |
| Fisher's Exact Test |  |  |  |
| Linear-by-Linear Association | 15.923 | 1 | .000 |
| N of Valid Cases | 340 |  |  |
| a. 0 cells (.0%) have expected count less than 5. The minimum expected count is 31.31. | | | |
| b. Computed only for a 2x2 table | | | |

| **Chi-Square Tests** | | |
| --- | --- | --- |
|  | Exact Sig. (2-sided) | Exact Sig. (1-sided) |
| Pearson Chi-Square |  |  |
| Continuity Correction^b^ |  |  |
| Likelihood Ratio |  |  |
| Fisher's Exact Test | .000 | .000 |
| Linear-by-Linear Association |  |  |
| N of Valid Cases |  |  |
|  | | |
| b. Computed only for a 2x2 table | | |

**muscosk_pain * City**

| **Crosstab** | | | | | |
| --- | --- | --- | --- | --- | --- |
|  | | | City | | Total |
|  |  |  | Rural | Urban |  |
| muscosk_pain | 0 | Count | 82 | 28 | 110 |
|  |  | % within City | 26.7% | 23.7% | 25.9% |
|  | 1 | Count | 225 | 90 | 315 |
|  |  | % within City | 73.3% | 76.3% | 74.1% |
| Total | | Count | 307 | 118 | 425 |
|  |  | % within City | 100.0% | 100.0% | 100.0% |

| **Chi-Square Tests** | | | |
| --- | --- | --- | --- |
|  | Value | df | Asymp. Sig. (2-sided) |
| Pearson Chi-Square | .395^a^ | 1 | .530 |
| Continuity Correction^b^ | .255 | 1 | .614 |
| Likelihood Ratio | .400 | 1 | .527 |
| Fisher's Exact Test |  |  |  |
| Linear-by-Linear Association | .394 | 1 | .530 |
| N of Valid Cases | 425 |  |  |
| a. 0 cells (.0%) have expected count less than 5. The minimum expected count is 30.54. | | | |
| b. Computed only for a 2x2 table | | | |

| **Chi-Square Tests** | | |
| --- | --- | --- |
|  | Exact Sig. (2-sided) | Exact Sig. (1-sided) |
| Pearson Chi-Square |  |  |
| Continuity Correction^b^ |  |  |
| Likelihood Ratio |  |  |
| Fisher's Exact Test | .621 | .309 |
| Linear-by-Linear Association |  |  |
| N of Valid Cases |  |  |
|  | | |
| b. Computed only for a 2x2 table | | |

**move_dis * City**

| **Crosstab** | | | | | |
| --- | --- | --- | --- | --- | --- |
|  | | | City | | Total |
|  |  |  | Rural | Urban |  |
| move_dis | No | Count | 7661 | 2904 | 10565 |
|  |  | % within City | 97.9% | 98.2% | 98.0% |
|  | Yes | Count | 167 | 54 | 221 |
|  |  | % within City | 2.1% | 1.8% | 2.0% |
| Total | | Count | 7828 | 2958 | 10786 |
|  |  | % within City | 100.0% | 100.0% | 100.0% |

| **Chi-Square Tests** | | | |
| --- | --- | --- | --- |
|  | Value | df | Asymp. Sig. (2-sided) |
| Pearson Chi-Square | 1.013^a^ | 1 | .314 |
| Continuity Correction^b^ | .866 | 1 | .352 |
| Likelihood Ratio | 1.038 | 1 | .308 |
| Fisher's Exact Test |  |  |  |
| Linear-by-Linear Association | 1.013 | 1 | .314 |
| N of Valid Cases | 10786 |  |  |
| a. 0 cells (.0%) have expected count less than 5. The minimum expected count is 60.61. | | | |
| b. Computed only for a 2x2 table | | | |

| **Chi-Square Tests** | | |
| --- | --- | --- |
|  | Exact Sig. (2-sided) | Exact Sig. (1-sided) |
| Pearson Chi-Square |  |  |
| Continuity Correction^b^ |  |  |
| Likelihood Ratio |  |  |
| Fisher's Exact Test | .360 | .176 |
| Linear-by-Linear Association |  |  |
| N of Valid Cases |  |  |
|  | | |
| b. Computed only for a 2x2 table | | |

**Parkinson * City**

| **Crosstab** | | | | | |
| --- | --- | --- | --- | --- | --- |
|  | | | City | | Total |
|  |  |  | Rural | Urban |  |
| Parkinson | No | Count | 50 | 21 | 71 |
|  |  | % within City | 29.9% | 38.9% | 32.1% |
|  | Yes | Count | 117 | 33 | 150 |
|  |  | % within City | 70.1% | 61.1% | 67.9% |
| Total | | Count | 167 | 54 | 221 |
|  |  | % within City | 100.0% | 100.0% | 100.0% |

| **Chi-Square Tests** | | | |
| --- | --- | --- | --- |
|  | Value | df | Asymp. Sig. (2-sided) |
| Pearson Chi-Square | 1.499^a^ | 1 | .221 |
| Continuity Correction^b^ | 1.116 | 1 | .291 |
| Likelihood Ratio | 1.467 | 1 | .226 |
| Fisher's Exact Test |  |  |  |
| Linear-by-Linear Association | 1.492 | 1 | .222 |
| N of Valid Cases | 221 |  |  |
| a. 0 cells (.0%) have expected count less than 5. The minimum expected count is 17.35. | | | |
| b. Computed only for a 2x2 table | | | |

| **Chi-Square Tests** | | |
| --- | --- | --- |
|  | Exact Sig. (2-sided) | Exact Sig. (1-sided) |
| Pearson Chi-Square |  |  |
| Continuity Correction^b^ |  |  |
| Likelihood Ratio |  |  |
| Fisher's Exact Test | .243 | .146 |
| Linear-by-Linear Association |  |  |
| N of Valid Cases |  |  |
|  | | |
| b. Computed only for a 2x2 table | | |
